# Correction: Bayesian Genome Assembly and Assessment by Markov Chain Monte Carlo Sampling

**DOI:** 10.1371/journal.pone.0104331

**Published:** 2014-07-28

**Authors:** The PLOS ONE Staff

## Notice of Republication

This article was republished on July 11th, 2014, due to Figure 4 and Figure 5 being ordered incorrectly. Please download the PDF again to view the corrected article. The originally published, uncorrected article and the republished, corrected article are provided here for reference.

## Supporting Information

File S1 Originally published, uncorrected article.
(pdf)

File S2 Republished, corrected article.
(pdf)
