# Supplementary material for: Correction: Bayesian Genome Assembly and Assessment by Markov Chain Monte Carlo Sampling
Source: PLoS One. 2014 Jul 28;9(7):e104331. doi: 10.1371/journal.pone.0104331 (PMC4113437; doi:10.1371/journal.pone.0104331)
Supplement: File S1 — Originally published, uncorrected article. (pdf) [file pone.0104331.s001.pdf]

OPEN ACCESS Freely available online

PLOS | ONE

# Bayesian Genome Assembly and Assessment by Markov Chain Monte Carlo Sampling

**Mark Howison[1]*, Felipe Zapata[2], Erika J. Edwards[2], Casey W. Dunn[2]**

**1** Center for Computation and Visualization, Brown University, Providence, Rhode Island, United States of America, **2** Department of Ecology and Evolutionary Biology, Brown University, Providence, Rhode Island, United States of America

## Abstract

Most genome assemblers construct point estimates, choosing only a single genome sequence from among many alternative hypotheses that are supported by the data. We present a Markov chain Monte Carlo approach to sequence assembly that instead generates distributions of assembly hypotheses with posterior probabilities, providing an explicit statistical framework for evaluating alternative hypotheses and assessing assembly uncertainty. We implement this approach in a prototype assembler, called Genome Assembly by Bayesian Inference (GABI), and illustrate its application to the bacteriophage ΦX174. Our sampling strategy achieves both good mixing and convergence on Illumina test data for ΦX174, demonstrating the feasibility of our approach. We summarize the posterior distribution of assembly hypotheses generated by GABI as a majority-rule consensus assembly. Then we compare the posterior distribution to external assemblies of the same test data, and annotate those assemblies by assigning posterior probabilities to features that are in common with GABI's assembly graph. GABI is freely available under a GPL license from https://bitbucket.org/mhowison/gabi.

**Citation:** Howison M, Zapata F, Edwards EJ, Dunn CW (2014) Bayesian Genome Assembly and Assessment by Markov Chain Monte Carlo Sampling. PLoS ONE 9(6): e99497. doi:10.1371/journal.pone.0099497

**Editor:** Magnus Rattray, University of Manchester, United Kingdom

**Received** February 4, 2014; **Accepted** May 15, 2014; **Published** June 26, 2014

**Copyright:** © 2014 Howison et al. This is an open-access article distributed under the terms of the Creative Commons Attribution License, which permits unrestricted use, distribution, and reproduction in any medium, provided the original author and source are credited.

**Funding:** This work was supported by the National Science Foundation (http://www.nsf.gov) through the Alan T. Waterman Award to CWD and award DEB-1026611 to EJE, and through additional support from the Brown Division of Biology and Medicine (http://biomed.brown.edu) to EJE. The funders had no role in study design, data collection and analysis, decision to publish, or preparation of the manuscript.

**Competing Interests:** The authors have declared that no competing interests exist.

* Email: mhowison@brown.edu

## Introduction

Most current methods for *de novo* genome assembly generate point estimates of the genome sequence without explicit statistical information about confidence in this particular estimate, or its support relative to other alternative assemblies supported by the same sequence data [1]. Recently, several probabilistic approaches have been proposed to quantify assembly certainty and address these limitations. Computing Genome Assembly Likelihood (CGAL) approximates the likelihood of an assembly given the sequence reads and a generative model learned from the data [2]; Log Average Probability (LAP) approximates the likelihood under a similar model of sequence read generation [3]; and Assembly Likelihood Evaluation (ALE) uses an empirical Bayesian approach to estimate the posterior probability of a particular assembly (or components of that assembly) [4]. Historically, the idea of applying statistical criteria to the fragment assembly problem was first proposed by Myers [5], who formulated the problem as a maximum-likelihood search.

Here we present an alternative Bayesian approach to approximating posterior probabilities, using Markov chain Monte Carlo (MCMC) sampling [6] to generate an entire posterior distribution of assembly hypotheses. An MCMC framework for sequence assembly addresses many of the general problems of accommodating assembly uncertainty, and provides a probabilistic understanding of the support for an assembly as a whole, as well as for particular features of the assembly that the investigator has special interest in. For example, it may be that each individual assembly hypothesis has low overall confidence, but that some feature, such as the order of a set of genes or existence of a specific regulatory element, is consistent across all the assembly hypotheses and has high confidence.

## Design and Implementation

To demonstrate the feasibility of the MCMC approach, we have implemented a prototype assembler called Genome Assembly by Bayesian Inference (GABI, https://bitbucket.org/mhowison/gabi). Because our approach is computationally expensive, our current tests are limited to the small but well-studied genome of the ΦX174 phage (which has NCBI Reference Sequencec NC_001422.1). Our input data consist of 2,000 read-pairs drawn from Illumina ΦX174 sample data (http://www.illumina.com/systems/miseq/scientific_data.ilmn). Aavailable as File S1, this subset of reads has maximum length 251 bp, an estimated mean insert size of 357 bp, and approximately 93× coverage of the ΦX174 genome.

Conveniently, our approach has technical parallels and similar conceptual advantages as the advances made over the past 15 years in the application of Bayesian approaches to phylogenetic analysis [7–9]. As in phylogenetics and other fields, designing an MCMC sampling strategy that achieves good mixing and convergence poses a variety of challenges and opportunities. For sequence assembly, the challenge is to design a mechanism for proposing new assemblies, a means of calculating the likelihood of a proposed assembly under an explicit model of read generation,

and the specification of a prior probability distribution that describes some prior beliefs about the assembly before the sequence data were collected. To implement an MCMC sequence assembler, we made the following choices:

### Proposal mechanism

We first build an assembly graph starting from a de Bruijn graph of the reads. Then we remove all tips (since the ΦX174 genome is circular) and merge all unambiguous paths into single nodes that are annotated by the sequence of merged $k$-mers. The resulting unresolved assembly graph (no longer de Bruijn) is a directed graph that consists only of bubbles and is a minimal representation of the variants that can be inferred from the sequenced data. Concatenating the sequences across the nodes in a particular path through this graph gives a possible assembly sequence.

A particular assembly hypothesis is represented as a boolean vector, whose values indicate which of the enumerated edges in the assembly graph are in an active path (Figure 1). We start with a random vector and propose new assemblies by toggling a value at random in the current vector. However, when toggling, we enforce the constraint that an active path cannot take multiple branches through the same bubble, since this would not spell a contiguous sequence. Instead, turning on an edge in an alternate branch turns on the *entire* alternate branch and turns off the existing path through the bubble (Figure 2). In this way, we can propose alternate paths through a complex bubble in one move of the sampler, without having to split the path and wait for the alternate branch to randomly activate. Furthermore, we can repeat this perturbation process a specified number of times for each new sample. Using several perturbations per sample causes the proposal mechanism to take larger steps through the combinatorial space of possible assemblies.

In our prototype implementation, we only consider paths that visit an edge at most once. In the general case, however, the proposal mechanism should be extended to accommodate paths that revisit edges, in order to resolve repeat regions of a genome.

### Likelihood

Because of the constraints described above, the active paths in every proposed assembly can be output as a FASTA file of concatenated node sequences. We run LAP directly on this FASTA file to estimate the proposed assembly's likelihood.

### Priors

An uninformative (e.g., flat) prior distribution could be used if nothing is known about the genome to be assembled. Since ΦX174 is well known, and it has previously been established that the genome is 5,386 bp and consists of one circular chromosome, we construct a prior probability distribution as the product of two gamma distributions, one for the sum of contig lengths (centered at 5,386) and the other for the number of contigs in the assembly (centered at 1). In other cases, external information about repeat structure or gene synteny could also be incorporated as priors.

## Results

To evaluate the mixing and convergence of our MCMC sampler, we ran three independent chains and compared their traces and split frequencies, as is common practice for other applications of MCMC [10]. Our results show that our design achieve d good mixing when using a simple Metropolis sampler [11] with three perturbations per assembly proposal (Figure 3a). The cumulative frequencies of the individual nodes and edges were mostly stabilized after 4,000 accepted samples (Figure 3b). Those with weak support were likely assembled from reads with sequencing errors. Other nodes and edges were more ambiguous, with a cumulative frequency hovering at an intermediate value or varying across samples. Ten nodes achieved a frequency of 100% (darkest red nodes in Figure 4a) and these include the four nodes representating the longest unambiguous sequences in the assembly graph (each longer than 400 bp), for a total of 2,428 bp of sequence.

Overall, the standard deviation in edge frequencies between the chains decrease d with additional sampling, indicating that the independent chains converged to the same posterior distribution (Figure 3c). The split frequencies among the chains were mostly correlated after the last sample (Figure 3d). We also tested the sampler with flat priors, with no likelihood calculations (priors only), and with different choices of the shape parameter for the gamma distributions (Figure 5). Our acceptance rate was 22.4%, which is close to the heuristic of 25% that can be considered optimal for general Metropolis sampling [12]. Aggregated across

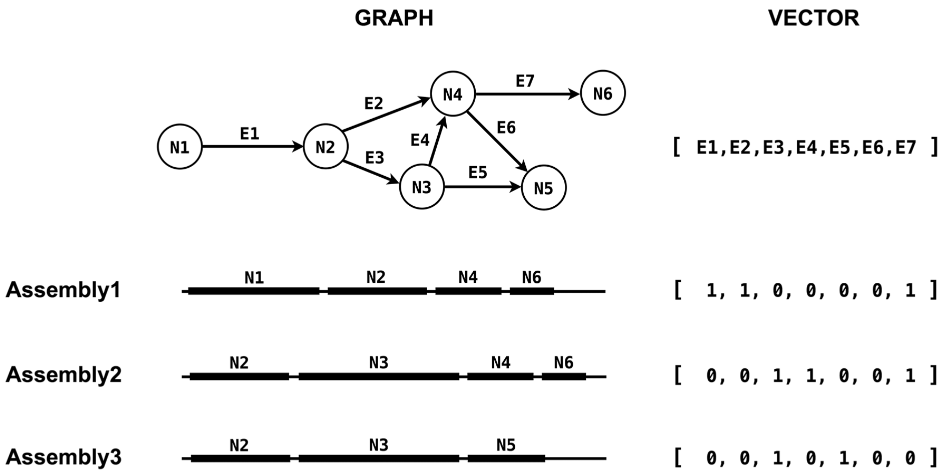


**Figure 1. Assembly representation.** Contiguous paths through the assembly graph correspond to proposed assemblies, and are represented by a boolean vector indicating which edges in the graph are active.
doi:10.1371/journal.pone.0099497.g001

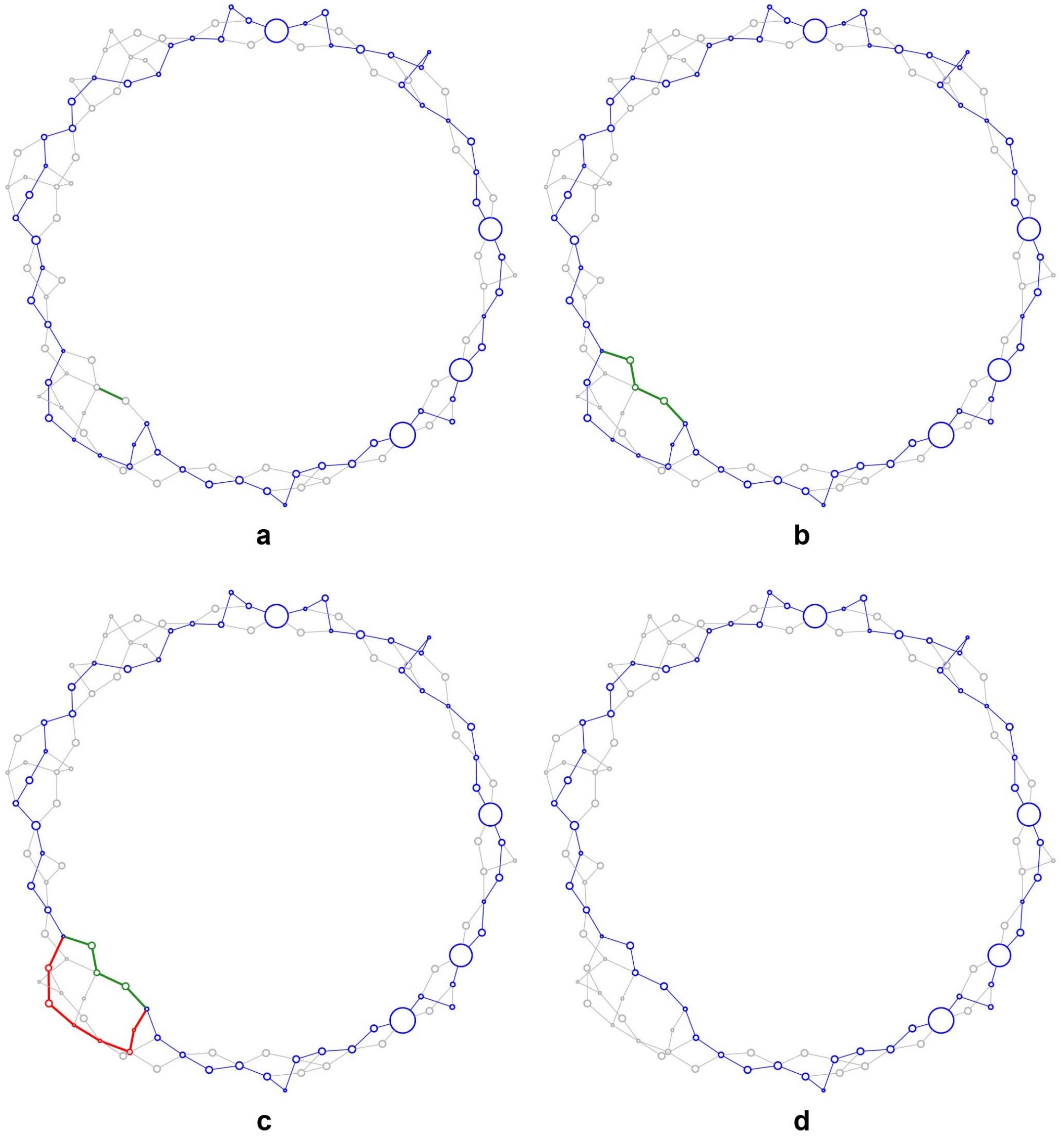


**Figure 2. Customized proposal mechanism.** (**a**) Starting from an existing (blue) assembly, the proposal mechanism randomly chooses a new (green) edge. (**b**) If the edge is not already active, it is extended with a random walk until it meets the existing assembly. (**c**) This defines a (red) branch in the existing assembly, (**d**) which is then removed to yield a new (blue) assembly.
doi:10.1371/journal.pone.0099497.g002

all three chains, the mean compute time per sample was 1.2 seconds and total compute time was 166.3 CPU-hours.

GABI provides multiple ways for summarizing the results of an MCMC analysis and is built on top of BioLite, a light-weight bioinformatics framework with rich diagnostics and reporting capabilities [13]. The assembly graph can be annotated with the approximated posterior probabilities (Figure 4a), or a consensus assembly can be extracted, for instance a majority-rule consensus that shows all edges occurring with frequency $>50\%$ (Figure 4b). The report also includes a FASTA file for the majority-rule consensus, annotated with the posterior probabilities of its components, and an interactive animation of the sampling for each MCMC chain, created with the D3js data visualization toolkit [14]. A sample report for the MCMC chains analyzed here is archived in the Brown Digital Repository [15].

GABI includes a tool to assign posterior probabilities to features of external assemblies that correspond to features in its own assembly graph. This provides an explicit and unified statistical framework for comparison of assemblies produced by multiple methods and software tools. Here, we compare NCBI Reference Sequence NC_001422.1 and *de novo* point-estimate assemblies generated by the String Graph Assembler [16] and Velvet [17]. We require exact matches to identify corresponding features, and this conservative strategy means that there is not an exact correspondence between the NCBI reference sequence and the GABI graph (Figure 4e). For this simple assembly problem, there is nearly universal agreement among the assemblies: both the majority rule consensus (Figure 4b) and NCBI reference sequence (Figure 4e) are proper subsets of the two *de novo* point-estimate assemblies (Figure 4c–d). The one notable difference is that the SGA assembly contains two contigs that choose alternate paths in

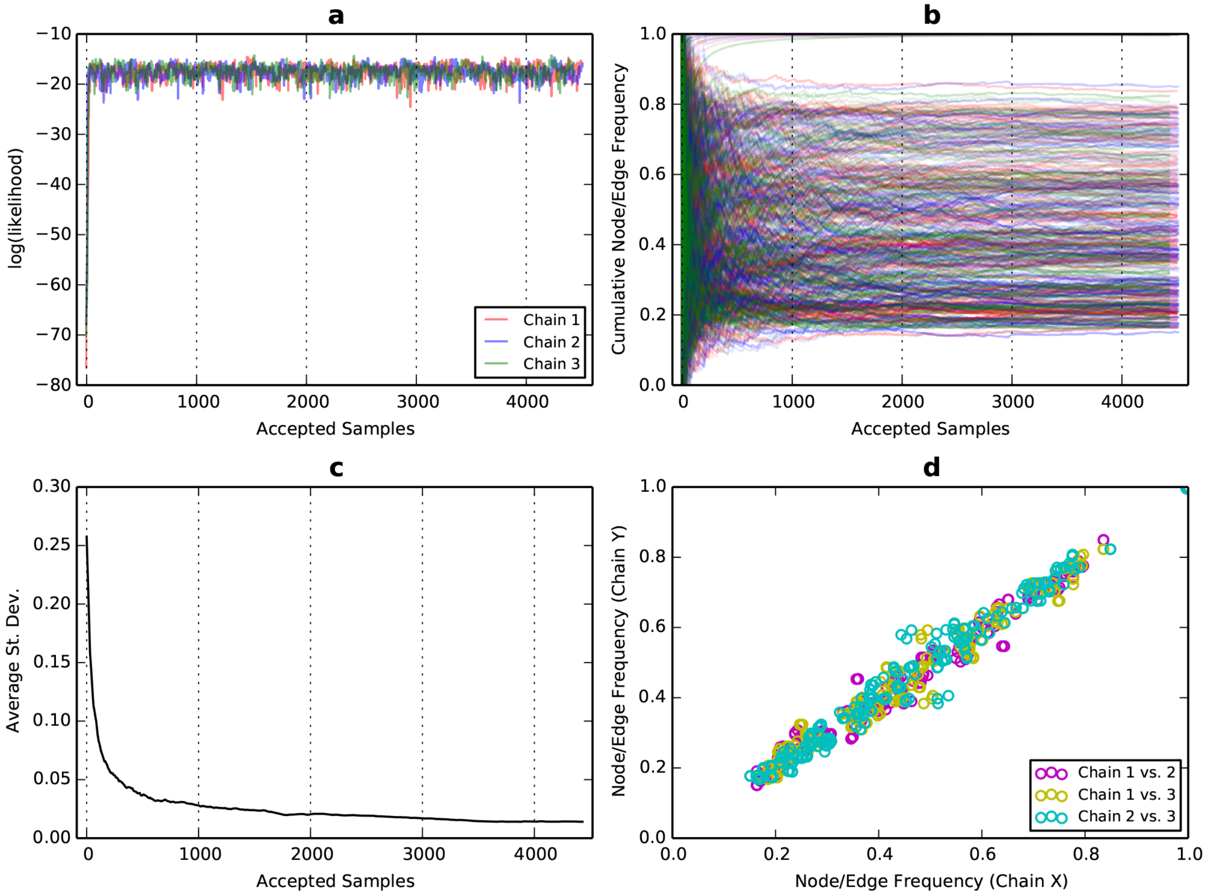


**Figure 3. Evidence of good mixing and convergence of three independent MCMC assembly chains.** (**a**) Early in the sampling, the log(likelihood) reaches a stationary distribution with random noise, indicating good mixing of the chains. (**b**) Plotting the cumulative node/edge frequencies shows that most of the frequencies have reached a stationary value. (**c**) The average standard deviation among the three chains of the cumulative frequencies approaches zero. (**d**) A bivariate plot comparing node/edge frequencies between each pair of chains shows that the frequencies are in agreement across all chains. Both (**c**) and (**d**) indicate convergence.
doi:10.1371/journal.pone.0099497.g003

one of the bubbles, and these alternate paths have similar posterior probabilities (green highlight in Figure 4c ).

## Discussion

A challenge to scaling MCMC assembly is that the combinatorial complexity of larger assembly graphs could become prohibitive for full *de novo* Bayesian assembly of large genomes. There are several ways to address this, such as applying more sophisticated sampling methods like Metropolis-coupled MCMC [18] or bridging states [19] that can explore larger combinatorial spaces more efficiently; constraining the assembly graph to focus on particular assembly hypotheses instead of attempting full *de novo* assembly; partitioning a larger genome based on an existing draft assembly and performing MCMC assembly concurrently on subgraphs of less complexity; or pruning the assembly graph using additional data from restriction site mappings [20]. Another promising application is transcriptome assembly, since the assembly graphs for individual transcripts should be less complex and can be sampled independently.

Our prototype assembler cannot yet consider repeats, as each edge can only be visited once. Repeats are common in many genomes, and addressing them is critical if this approach is to be widely adopted. Fortunately, a straight-forward extension of our graph representation from a vector space of booleans to a vector space of nonÂ-negative integers can represent repeats, where the integer value for an edge denotes how many times it is visited. The proposal mechanism can be modified to enforce constraints on the in- and out-degree of the nodes in the path, to ensure that a proposed path can be unwound into a valid, contiguous sequence. Because paired-end alignments are used to calculate the likelihood, a path with the correct number of repeats will score a higher likelihood. We expect this to work well because two maximum-likelihood assembly approaches have already used the same principle to demonstrate high accuracy in resolving repeat regions [21,22].

Like existing assemblers, GABI can be used to assemble a point estimate of a genome, but unlike most other assemblers, the resulting assembly will have been chosen according to explicit statistical criteria (posterior probability) and will have associated information on the confidence in various aspects of its sequence and structure. In addition, MCMC assembly provides new opportunities for investigators who are interested not only in the certainty of a particular inference (e.g. an ALE [4] estimate of posterior probability for a given assembly), but in the many alternative hypotheses that are also supported by the data. The

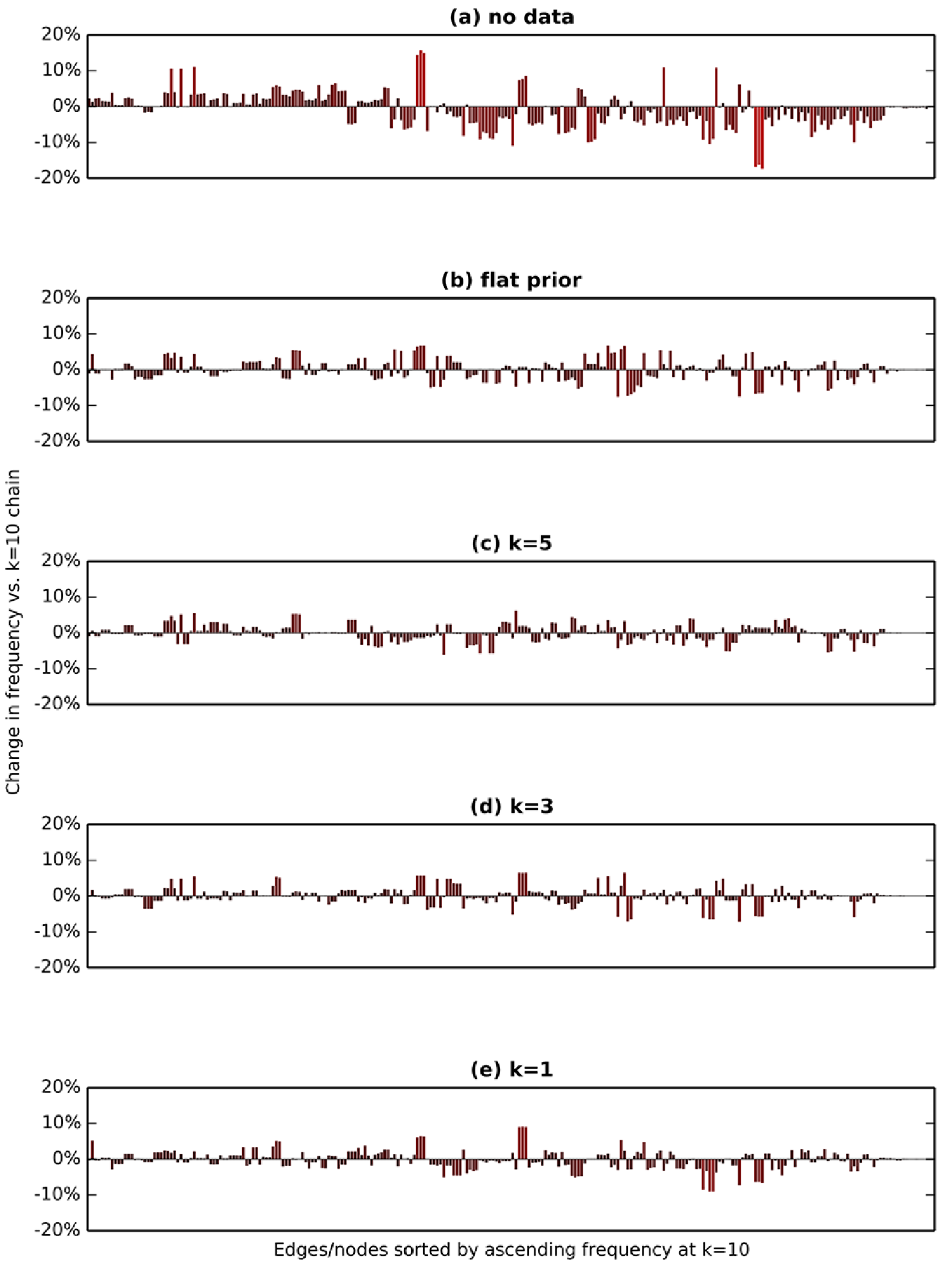


**Figure 4. Graphical summaries and comparisons of the ΦX174 genome assemblies.** Each node is a sequence fragment, and alternative paths through the graph represent alternative assemblies. Node size is proportional to the log length of its sequence. (**a**) Posterior probabilities for each node/edge (dark red indicates a posterior of 1.0, grey a posterior of 0.0 ). (**b–e**) Components of the GABI assembly graph that are found exactly in the majority-rule consensus GABI assembly (**b**), SGA assembly (**c**), Velvet assembly (**d**), and NCBI reference sequence (**e**).
doi:10.1371/journal.pone.0099497.g004

MCMC approach addresses many of the general problems of summarizing assembly uncertainty and will allow assembly uncertainty to be propagated to downstream analyses [1].

## Methods

The results presented were generated with GABI version 0.2.0 and are recomputable using included scripts (https://bitbucket.org/mhowison/gabi/src/master/phix-test). Here, we provide brief comments on the technical details.

### Compute resources

All tests were run at the Center for Computation and Visualization, Brown University, on IBM iDataPlex nodes, each equipped with 8-core, dual-socket Intel Xeon E5540 (2.53 Ghz) processors and 24 GB of memory. CPU-hours were calculated as total walltime across all nodes, multiplied by 8 CPUs per node.

### Subset of ΦX174 sample data

We started with 8.36 Gb of paired-end reads of maximum length 251 bp from Illumina ΦX174 sample data (ftp://webdata:webdata@ussd-ftp.illumina.com/Data/SequencingRuns/

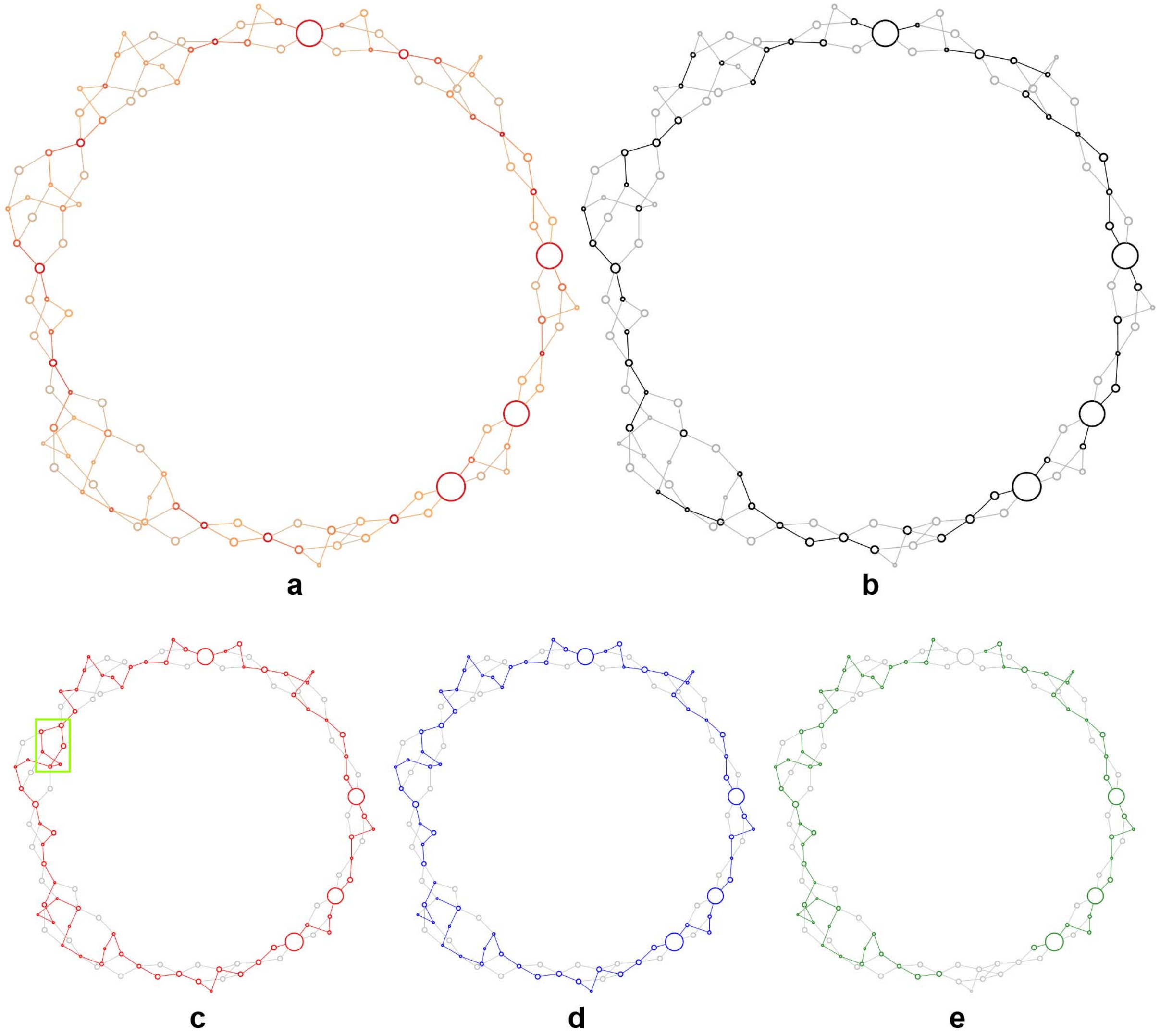


**Figure 5. The prior probabilities influence the posterior distribution.** Running the sampler with no data so that the posterior probabilities are determined by the prior probabilities only (**a**), with flat priors (**b**), or with different values of the shape parameter $k$ for the gamma distributions of genome length and number of contigs (**c–e**) all cause shifts in the posterior distribution of edge frequencies. The baseline for the change in frequency is Chain 1 from Figure 3 with $k=10$, and all chains here were initialized with the same random seed as Chain 1.
doi:10.1371/journal.pone.0099497.g005

PhiX/PhiX_S1_L001_R1_001.fastq.gz and ftp://webdata:webdata@ussd-ftp.illumina.com/Data/SequencingRuns/PhiX/PhiX_S1_L001_R2_001.fastq.gz, accessed 2013 Apr 26). Using the bl-filter-illumina tool from BioLite, we chose the first 2,000 read pairs that did not contain known Illumina adapter sequences and that had mean quality score greater than 37 (Phred-33 scoring). This procedure is available in the script https://bitbucket.org/mhowison/gabi/src/master/phix-test/00-subset-data.sh.

## SGA and Velvet assemblies

To assemble the ΦX174 subset with SGA, we followed the example provided for an assembly of E. coli from similar MiSeq reads (https://github.com/jts/sga/blob/master/src/examples/sga-ecoli-miseq.sh). To assemble with Velvet, we used the included VelvetOptimiser tool to sweep coverage and cutoff parameters for a $k$-mer size of 99. The estimated mean and standard deviation of the insert size reported earlier were obtained from VelvetOptimiser's output. This procedure is available in the script https://bitbucket.org/mhowison/gabi/src/master/phix-test/01-assemble.sh.

## De Bruijn graph reduction

First, we recursively and completely trimmed all tips (edges with an incidence of one), because the ΦX174 genome is circular (for a linear genome, a softer tip trimming threshold would be more appropriate). Next, we merged all simple paths through the graph, similar to the process of reducing an *overlap graph* to a *string graph* [23]. We annotated the new node with the accumulated overlap of the merged nodes' $k$-mers, which accumulates one additional nucleotide for each merged node. Finally, we split the graph into weakly connected components and choose the largest one. A path of edges through the graph spells a contig by concatenating the annotations on the nodes in the path.

### Odds ratio

To determine whether to accept a proposed assembly hypothesis, we calculate the odds ratio [11] of the posterior probabilities of the old assembly, $P(H|D)$, and the new perturbed assembly, $P(H^*|D)$, as:

$$R = \frac{P(H^*|D)}{P(H|D)} = \frac{\frac{P(D|H^*)P(H^*)}{P(D)}}{\frac{P(D|H)P(H)}{P(D)}} = \frac{P(D|H^*)P(H^*)}{P(D|H)P(H)} \quad (1)$$

If this ratio is greater than 1, the new assembly is automatically accepted. If it is less than 1, it is accepted with probability R. The process is then repeated until there is a stationary distribution of assemblies in the sample, which occurs when the frequency of assembly attributes does not change with additional sampling.

## Supporting Information

**File S1 ΦX174 sequence data.** A subset of 2,000 paired-end reads (maximum length 251 bp, estimated mean insert size 357 bp) drawn from Illumina MiSeq sample data (http://www.illumina.com/systems/miseq/scientific_data.ilmn ).
(ZIP)

## Acknowledgments

This research was conducted using computational resources and services at the Center for Computation and Visualization, Brown University.

## Author Contributions

Conceived and designed the experiments: MH FZ CWD EJE. Performed the experiments: MH. Analyzed the data: MH. Contributed reagents/materials/analysis tools: MH. Wrote the paper: MH FZ CWD.

## References

1. Howison M, Zapata F, Dunn CW (2013) Toward a statistically explicit understanding of de novo sequence assembly. Bioinformatics 29: 2959–2963. doi:10.1093/bioinformatics/btt525.
2. Rahman A, Pachter L (2013) CGAL: computing genome assembly likelihoods. Genome Biology 14: R8. doi:10.1186/gb-2013-14-1-r8.
3. Ghodsi M, Hill CM, Astrovskaya I, Lin H, Sommer DD, et al. (2013) De novo likelihood-based measures for comparing genome assemblies. BMC Research Notes 6: 334. doi:10.1186/1756-0500-6-334.
4. Clark SC, Egan R, Frazier PI, Wang Z (2013) ALE: a generic assembly likelihood evaluation framework for assessing the accuracy of genome and metagenome assemblies. Bioinformatics 29: 435–443. doi:10.1093/bioinformatics/bts723.
5. Myers EW (1995) Toward simplifying and accurately formulating fragment assembly. Journal of Computational Biology 2: 275–290. doi:10.1089/cmb.1995.2.275.
6. Gilks WR, Richardson S, Spiegelhalter D (1995) Markov Chain Monte Carlo in Practice. London: Chapman and Hall/CRC, 1 edition.
7. Holder MT, Lewis PO (2003) Phylogeny estimation: traditional and Bayesian approaches. Nature Reviews Genetics 4: 275–284. doi:10.1038/nrg1044.
8. Ronquist F, Huelsenbeck JP (2003) MrBayes 3: Bayesian phylogenetic inference under mixed models. Bioinformatics 19: 1572–1574. doi:10.1093/bioinformatics/btg180.
9. Lartillot N, Lepage T, Blanquart S (2009) PhyloBayes 3: a Bayesian software package for phylogenetic reconstruction and molecular dating. Bioinformatics 25: 2286–2288. doi:10.1093/bioinformatics/btp368.
10. Nylander JAA, Wilgenbusch JC, Warren DL, Swofford DL (2008) AWTY (are we there yet?): a system for graphical exploration of MCMC convergence in Bayesian phylogenetics. Bioinformatics 24: 581–583. doi:10.1093/bioinformatics/btm388.
11. Matropolis N, Rosenbluth AW, Rosenbluth MN, Teller AH, Teller E (1953) Equation of State Calculations by Fast Computing Machine. Journal of Chemical Physics 21: 1087–1092. doi: doi:10.1063/1.1699114.
12. Gelman A, Gilks WR, Roberts GO (1997) Weak convergence and optimal scaling of random walk Metropolis algorithms. The Annals of Applied Probability 7: 110–120. doi:10.1214/aoap/1034625254.
13. Howison M, Sinnott-Armstrong NA, Dunn CW (2012) BioLite, a lightweight bioinformatics frame-work with automated tracking of diagnostics and provenance. In: Proceedings of the 4th USENIX Workshop on the Theory and Practice of Provenance (TaPP'12). Available: https://www.usenix.org/conference/tapp12/workshop-program/presentation/Howison. Accessed 2014 May 27.
14. Bostock M, Ogievetsky V, Heer J (2011) $D^3$: Data-driven documents. IEEE Transactions on Visualization and Computer Graphics 17: 2301–2309. doi:10.1109/TVCG.2011.185.
15. Howison M (2014). Genome Assembly by Bayesian Inference (GABI): Sample Report for PhiX174. Brown Digital Repository. Available: https://repository.library.brown.edu/viewers/archive/bdr:351764. Accessed 2014 May 27. doi:10.7301/Z0H41PB7.
16. Simpson JT, Durbin R (2012) Efficient de novo assembly of large genomes using compressed data structures. Genome Research 22: 549–556. doi:10.1101/gr.126953.111.
17. Zerbino DR, Birney E (2008) Velvet: Algorithms for de novo short read assembly using de Bruijn graphs. Genome Research 18: 821–829. doi:10.1101/gr.074492.107.
18. Geyer CJ (1991) Markov chain Monte Carlo maximum likelihood. In: Proceedings of the 23rd Symposium on the Interface. 156–163.
19. Lin D, Fisher J (2012) Efficient Sampling from Combinatorial Space via Bridging. In: Proceedings of the 15th International Conference on Artificial Intelligence and Statistics (AISTATS 2012). 694–702.
20. Lam ET, Hastie A, Lin C, Ehrlich D, Das SK, et al. (2012) Genome mapping on nanochannel arrays for structural variation analysis and sequence assembly. Nature Biotechnology 30: 771–776. doi:10.1038/nbt.2303.
21. Medvedev P, Brudno M (2009) Maximum likelihood genome assembly. Journal of Computational Biology 16: 1101–1116. doi:10.1089/cmb.2009.0047.
22. Varma A, Ranade A, Aluru S (2011) An improved maximum likelihood formulation for accurate genome assembly. In: Proceedings of the 1st IEEE International Conference on Computational Advances in Bio and Medical Sciences (ICCABS). 165–170. doi:10.1109/ICCABS.2011.5729873.
23. Myers EW (2005) The fragment assembly string graph. Bioinformatics 21: ii79–ii85. doi:10.1093/bioinformatics/bti1114.
